# The autophagy gene product BEC-1 supports normal aging and neurodevelopment in *Caenorhabditis elegans* II

**DOI:** 10.17912/micropub.biology.000100

**Published:** 2019-06-14

**Authors:** Nicholas Ashley, Andrea M Holgado

**Affiliations:** 1 St. Edward's University, Department of Biological Sciences, Austin, TX 78704, USA

**Figure 1.**
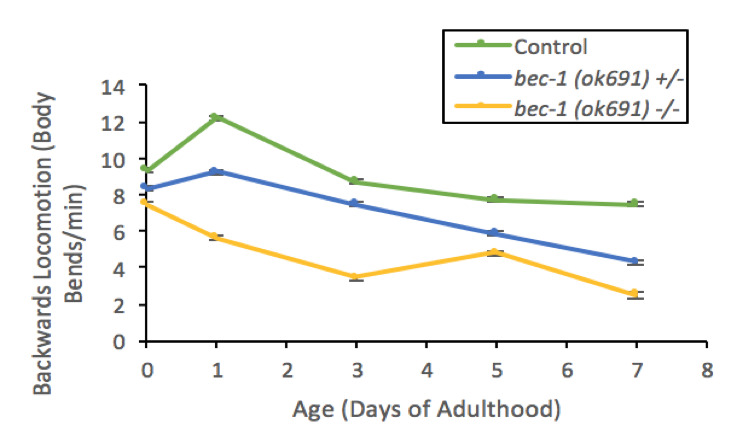
**Homozygous *bec-1(ok691)* mutants show defects in backwards locomotion in response to nose touch.** Backwards locomotion rate throughout days 0,1,3,5, and 7 of adulthood was measured. Data plotted are mean ± 1 SEM, n=60. Statistical analysis was performed using a non-parametric Kruskal-Wallis (p< .001).

## Description

Macroautophagy (hereon referred as Autophagy) is a cellular housekeeping mechanism that uses a double membrane to target and engulf cell products forthe formation of autophagosomes. These double membrane organelles then fuse to lysosomes where cell products aredegraded and recycled (Nakamura and Yoshimori, 2018). The loss of autophagy function in the motor cortex has been associated with progression of neurodegenerative symptoms in Parkinson’s disease (Kaila and Lang 2015; Fahn et al. 2004). To better understand the role of Beclin 1 and autophagy in neurons, we studied locomotion in *C. elegans* mutants for *bec-1*. Locomotion in both homozygous (*bec-1* -/-) and heterozygous *bec-1(ok691)* (*bec-1* +/-) mutants showed significant defects (Figure 1). Homozygous *bec-1(ok691)* mutants backwards locomotion rates ranged between 3-8 body bends/minute at ages 1-7 days of adulthood and heterozygous *bec-1(ok691)* mutants were 6-9 body bends/minute at comparable ages. These defects in locomotion suggest a role of autophagy controlling behaviors that are neuronal dependent.

Previous research has shown that loss of motor function could be a result of neurodegeneration (Fahn et al., 2004; Kalia and Lang, 2015). Additionally, *C. elegans* studies demonstrated that mutations in *bec-1* associate with degeneration of muscle cells and aging (Herndon et al., 2002). Together, our findings and previous reportsindicate that autophagy gene product, BEC-1, may play an important role in motor neuron physiology, survival, or both. Jia and colleagues have observed collections of visible protein aggregates in nematodes that elicit locomotion defects (Jia et al., 2007). This conclusion should be considered as preliminary as we have not verified by an alternative line of investigation (e.g., a second allele or transgene rescue) that the observed phenotypes are specific to *bec-1(ok691).* However, our study is also consistent with previous findings in Parkinson’s disease patients where accumulation of protein aggregates was associated with loss of autophagy and accelerated onsets symptoms of decreased motor function (Ermine et al., 2018; Fahn et al., 2004).

## Methods

Synchronizing:

Mixed stage nematodes grown on NGM plates at 20 °C were floated off using 1 mL of M9 reagent and collected in 1.5 mL tubes. Tubes containing animals were centrifuged at 9.3 × g for 1 minute. After centrifugation, the supernatant was discarded and the worm pellet was kept and treated with 1 mL of Alkaline Bleach (2.0% bleach (VWR), 0.5N NaOH) for 7 minutes at room temperature with occasional mixing. Once the 7-minute treatment concluded, bleached animals were centrifuged at 9. 3 × g for 2 minutes to collect eggs. Pelleted eggs were washed 3 times with 1 mL of M9 and centrifuged for 1 min. at 9.3 × g. After centrifugation, the supernatant was discarded and the pelleted eggs were suspended. Two drops of resuspended eggs were placed onto seeded NGM plates.

Locomotion:

Individual synchronous nematodes were transferred to seeded NGM plates on days 0, 1, 3, 5, and 7 of adulthood. Locomotion was measured after a nose touch stimulation and backwards body bends were counted for 20 seconds. Backwards locomotion rate was reported as body bends per minute.

## Reagents

Strains CZ1200 and VC517 were obtained from the *C. elegans* Genetics Center. CZ1200 contains the transgene *juIs76*[*unc25p*::GFP] which drives the expression of GFP in d-type motor neurons. Strain AMH50 was produced in our laboratory by crossing CZ1200 with VC517 *bec-1(ok691)*/*nT1*[*qIs51*]. AMH50 possess the balanced lethal mutation *bec-1(ok691)*/*nT1*[*qIs51*] and the transgene *juIs76*, {*bec-1(ok691)*IV/*nT1*[*qIs51*] (IV;V); *juIs76*[*unc-25p*::GFP] II}.
